# Current Concepts in the Management of Postoperative Nausea and Vomiting

**DOI:** 10.1155/2011/748031

**Published:** 2011-11-03

**Authors:** S. Chatterjee, A. Rudra, S. Sengupta

**Affiliations:** ^1^Department of Anaesthesiology, Medical College & Hospital, 88 College Street, Kolkata, India; ^2^Department of Anaesthesiology, K.P.C Medical College, Jadavpur, Kolkata, India; ^3^Department of Anaesthesiology, Apollo Gleneagles Hospital, 58 Canal Circular Road, Kolkata, India

## Abstract

Postoperative nausea and vomiting (PONV) are still common following surgery. This is not only distressing to the patient, but increases costs. The thorough understanding of the mechanism of nausea and vomiting and a careful assessment of risk factors provide a rationale for appropriate management of PONV. Strategy to reduce baseline risk and the adoption of a multimodal approach will most likely ensure success in the management of PONV.

## 1. Introduction

Postoperative nausea and vomiting (PONV) are two of the most common and unpleasant side effects following anaesthesia and surgery. The overall incidence of PONV has decreased from 60% when ether and cyclopropane were used, to approximately 30% at present [1]. However, in certain high-risk patients this incidence is still as high as 70%. It is estimated that an episode of vomiting prolongs postanaesthetic care unit (PACU) stay by about 25 minutes [2]. Patients not only rank the absence of PONV as being important [3], but also rank it more important than an earlier discharge from an ambulatory surgical unit [4]. In one survey, patients were willing to pay at their own expense, for a completely effective antiemetic [5]. Furthermore, it is estimated that approximately 0.2% of all patients may experience intractable PONV, leading to a delay in recovery room discharge and/or unanticipated hospital admission following ambulatory surgery, thereby increasing medical costs. Recent interest has focused on the use of a combination of antiemetics acting at different receptors and the adoption of a multimodal approach to tackle this problem. This paper will discuss the pathophysiology and risk factors of PONV, the use of multimodal approach, and novel therapy of PONV management. Finally, recommendations for the prophylaxis and treatment of PONV will also be discussed. 

## 2. Anatomy

**Figure 1:**
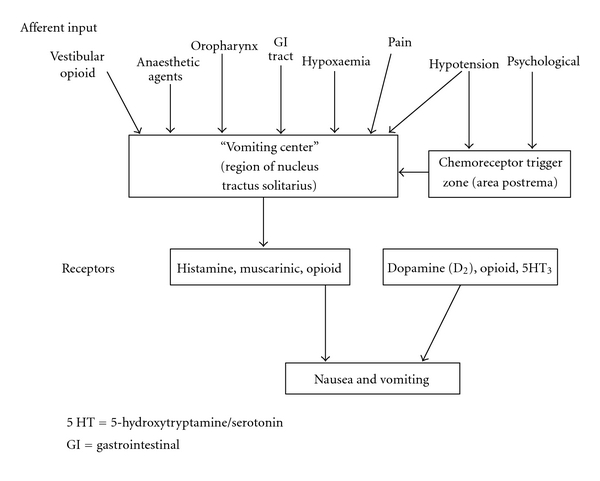
The inputs and receptors involved in causing PONV. 5 HT: 5- hydroxytryptamine/serotonin; GI: gastrointestinal.

The neuroanatomical site controlling nausea and vomiting is an ill-defined region called the “vomiting center” within the lateral reticular formation in the brainstem. The vomiting center receives afferent inputs from higher cortical centers, the cerebellum, the vestibular apparatus, and vagal and glossopharyngeal nerves. Further interactions occur with the nucleus tractus solitarius and the chemoreceptor trigger zone (CTZ) which is located in the floor of the fourth ventricle. The CTZ is outside the blood-brain barrier and in contact with cerebrospinal fluid (CSF). The CTZ enables substances in the blood and CSF to interact. Direct stimulation of the CTZ does not result in vomiting. Immunochemical studies of these anatomical sites show that these areas contain histamine, serotonin, cholinergic, neurokinin-1, and D2 dopamine receptors (Figure 1). 

## 3. Physiology [6]

The “vomiting reflex” is precipitated by different stimulation from the glossopharyngeal, hypoglossal, and vagal nerves reaching the vomiting center. Efferent signals are directed to the glossopharyngeal, hypoglossal, trigeminal, accessory, and spinal segmental nerves.There is a coordinated contraction of abdominal muscle against a closed glottis, which raises intra-abdominal and intrathoracic pressures. The pyloric sphincter contracts and the oesophageal sphincter relaxes, and there is active antiperistalsis within the oesophagus, which forcibly expels the gastric contents. This is associated with marked vagal and sympathetic activity leading to sweating, pallor, and bradycardia. 

## 4. Who Is at Risk for Postoperative Nausea and Vomiting 

The modern era in PONV risk factor research began in the early 1990s, with publication of the initial studies that attempted to simultaneously identify multiple risk factors. The identification of individual at high risk for PONV can narrow the pool of potential candidates for prophylactic antiemetic therapy, indicating those most likely to benefit and reducing antiemetic side effects and costs for patients unlikely to benefit.

## 5. In Adults

Only a few risk factors have consistently been shown to be independent predictors for PONV [7–12].

### 5.1. *Patient-Related Independent Predictors*

#### 5.1.1. Female Gender

The reason for increased female susceptibility to nausea and vomiting is not clear. PONV increases during menstruation and preovulatory phase of the menstrual cycle due to sensitization of the chemoreceptor trigger zone (CTZ) and vomiting center to follicle-stimulating hormone (FSH) and oestrogen. However, this gender difference in PONV is not noticed in paediatric age group and population aged more than 60 years.

#### 5.1.2. Nonsmoking

Cohen et al. were the first to determine that nonsmokers are almost twice as likely as smokers to have PONV [12]. Several other workers [13–16] have validated that findings of Cohen et al. Chronic exposure to smoke (particularly the polycyclic aromatic hydrocarbons) produces changes in liver microsomal enzymes that may affect the metabolism of drugs used in the perioperative period and the ability of these drugs to produce PONV. The protective aspect of smoking on postoperative nausea and vomiting is not likely to be attributable to an acute action of smoke constituents.

#### 5.1.3. History of PONV, Motion Sickness, or Migraine

Susceptibility to emetogenic stimuli increases among individuals with a personal history of PONV [10, 11], motion sickness, or migraine [13–17].

#### 5.1.4. Age

Among pediatric patients the incidence has been shown to be as high as 34% in the 6–10 year age group but considerably lower in younger patients, and the incidence decreases with the onset of puberty. In adults, the incidence of PONV appears to decrease with age [12, 15, 17, 18].

#### 5.1.5. Obesity

A body mass index of more than 30 in patients had been associated with PONV. This may be due to an increased intra-abdominal pressure and the pharmacokinetic effects of lipophilic anesthetic agents having prolonged half-lives in these patients. However, recent data [19] suggests that BMI is not correlated with an increased risk for the development of PONV. An increased BMI may increase the incidence of PONV in patients with other independent risk factors.

### 5.2. *Anaesthesia-Related Independent Predictors*

#### 5.2.1. Postoperative Opioids

Most larger studies demonstrate that use of postoperative opioids approximately doubles the risk of PONV [7, 11, 14]. The total dose of postoperative opioid [20], but not the particular type [21], appears to be of relevance. However, opioid given to a patient already in pain is not associated with significant increase in PONV episodes. Ultra short-acting opioid-like remifentanil has been found to have similar incidences of PONV as fentanyl over the first 24 hour postoperative period [14, 22].

#### 5.2.2. Inhalational Anaesthetics

There were no differences in incidence of PONV among the individual volatile anaesthetics (comparing halothane, isoflurane, sevoflurane, and desflurane) at 1 MAC or below [23–25]. However, volatile induction maintenance anaesthesia (VIMA) is associated with lesser PONV than balanced anaesthesia using opioids. Apfel and colleagues [9] have demonstrated that volatile anaesthetics are the main cause of PONV within the first two postoperative hours.

#### 5.2.3. Nitrous Oxide (N_2_O)

The emetogenic effect of nitrous oxide has received considerable attention in the literature with numerous studies in the 1980s and meta-analyses in the 1990s emphasizing the increased incidence of PONV with this agent [26]. However, in practice, the emetogenic effects of nitrous oxide and volatile anaesthetics are independent. that is, they are additive and not synergistic overlapping [14]. Bivariate analysis indicated that substituting propofol for a volatile anesthetic reduced the risk of postoperative nausea and vomiting by about 19 percent, whereas substituting nitrogen for nitrous oxide reduced the risk by about 12 percent [14]. In a prospective randomized study of 2050 patients avoidance of nitrous oxide and the concomitant increase in inspired oxygen concentration decreases the incidence of complications after major surgery, but does not significantly affect the duration of hospital stay [27]. A recent meta-analysis demonstrated an overall reduction in risk of PONV of 20% by avoiding N_2_O, the absolute difference in the incidence of PONV between the two groups is however small (33% with N_2_O and 27% without N_2_O) [28].

#### 5.2.4. Duration of Anaesthesia

The effect of increasing duration of anaesthesia on PONV is described in a number of studies [10, 16, 23]. The incidence may be increased when inhalational anaesthetics are used and decreased when propofol is used and in addition depends on the amount of opioids given. Increasing the operative duration by 30 minutes may increase the risk of PONV by 60%.

### 5.3. Surgery-Related Independent Predictors

 Although type of surgery has been identified as a risk factor in numerous reports, its status as such is still somewhat controversial; the specific procedures implicated as particularly emetogenic sometimes vary among studies. Types of procedures that may be viewed as possible risk factors include intraabdominal, laparoscopic, orthopaedic, major gynaecological, ear nose and throat (ENT), thyroid, breast, and plastic surgery as well as neurosurgery. High rate of PONV in laparoscopy may be caused by the gas used to “inflate” the abdomen to create work place for the instruments. This puts pressure on the vagus nerve, which has a connection to the brain's nausea and vomiting center. In addition to this, patients undergoing day case gynaecological laparoscopy have a number of other risk factors for PONV, as female gender, use of perioperative opioid, and a journey home which is likely to lower the threshold to motion-induced emesis [29]. There was no significant difference between the risk of PONV after laparoscopic versus open cholecystectomy, and the effect of laparoscopy remained insignificant after risk adjustment in a generalized linear regression model [30].

### 5.4. Other Factors

High levels of anxiety and postoperative pain, especially of pelvic or visceral origin, may also lead to a higher incidence of postoperative nausea and vomiting.

## 6. In Paediatrics

Paediatric patients are not spared from postoperative vomiting, with peak incidence in schoolchildren of 34% to 50% [31]. In this population, only vomiting is reported due to difficulties in eliciting nausea in the young age group. It is one of the leading postoperative complaints from parents and the leading cause of readmission. Prior to puberty, gender differences for postoperative vomiting (POV) have not been identified [32, 33]. Operations associated with a high incidence of postoperative vomiting in children include strabismus, adenotonsillectomy, hernia repair, orchidopexy, and penile surgery [34]. Other risk factors for POV in children are the same as those in adults, with several important differences, POV increases as children grow up. It is rare in children younger than 2 years old. However, children aged more than 3 years have an average vomiting incidence of ≥40%. The increased vomiting incidence tapers when children reach puberty. Sex differences in risk of vomiting in children include adenotonsillectomy, strabismus repair, hernia repair, orchiopexy, and penile surgery [35].

Nonsteroidal anti-inflammatory drugs (NSAIDS) such as ketorolac, as well as paracetamol which have a central mode of action can reduce the need for opioids. Also, a multimodal approach combining reduced dosages of narcotics and NSAIDs allows potentiation of analgesic effect and decreased severity of complications from both groups. Use of rectal acetaminophen and regional anaesthesia techniques (e.g., caudal epidural) in the paediatric population decrease the use of perioperative opioids and consequently the incidences of PONV.

Regarding use of antiemetics in the paediatric population, efficacy appears comparable to that known for adult. There are few thoroughly conducted dose-response studies for antiemetics in adults, however, and even fewer in children. Thus, most paediatric doses are somewhat arbitrarily set at a fraction (1/5th to 1/25th) of the common adult dose [31].

## 7. Postdischarge Nausea and Vomiting (PDNV)

In our country, significant numbers of surgeries are now performed on an outpatient basis. In addition to being a major cause for lengthened stay and unanticipated admission, PDNV may also pose a significant problem to the patient after discharge [36]. Numerous studies and consensus guidelines have been published on prevention of PONV, but few have evaluated the efficacy of prophylaxis on PDNV or its impact on quality of living during recovery [1, 7, 23, 29]. Early ambulation has been reported to be a contributor to early emetic symptoms. Reported postdischarge nausea (PDN) incidences varied from 0% to 55% and postdischarge vomiting (PDV) incidences from 0% to 16% [23, 35–39]. However, it is not clear whether the risk factors for PDNV are the same as for PONV, or whether PONV in the postanaesthesia care unit (PACU) predicts PDNV [37, 38]. Carroll et al. reported that outpatients who were discharged home often chose to wait for resolution of emetic symptoms rather than to contact their physicians for antiemetic treatment [38]. Patient with PDNV are significantly more likely to have problems performing activities of living, have a lower satisfaction score, and higher negative economic impact than are those not experiencing PDNV [38].

Shorter-acting drugs are not as effective, especially when used at the minimally effective dose, and that antiemetics with a longer duration of action seem favourable. Therefore, dexamethasone, transdermal scopolamine, palonosetron, and the NK1-receptor antagonists may be reasonable first choices for the prevention of postdischarge nausea and vomiting [40].

## 8. Risk Factor Findings

### 8.1. Scoring System

**Figure 2:**
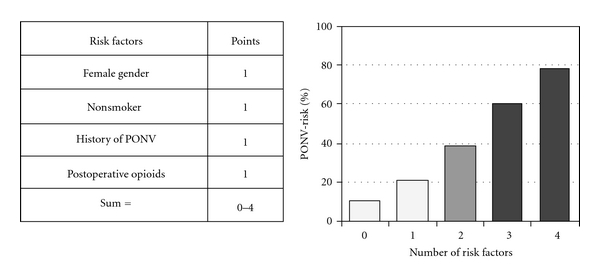
Simplified risk score for PONV in adults. (Reproduced from the original article by Gan et al. [52]).

A number of PONV risk scoring systems have been developed. Using logistic regression analysis by Palazzo and Evans, Koivuranta et al. generated a score based on the predictive factors [41, 42]. Recently, Apfel et al. developed a simplified risk score consisting of four predictors [7]. In adults, female gender, history of motion sickness or PONV, nonsmoking status, and the use of opioids for intraoperative or postoperative analgesia are used as risk factors and 1(one) point against each factor (Figure 2). 

**Figure 3:**
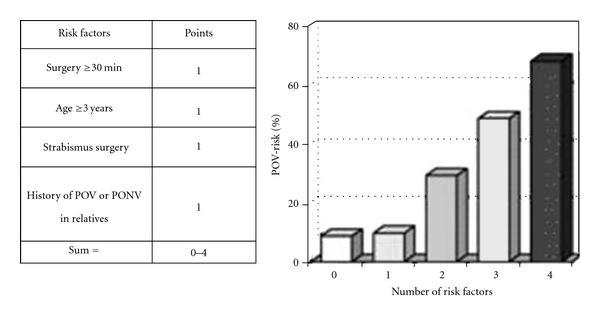
Simplified risk score for POV in children. (Reproduced from the original article by Gan et al. [52]).

In children, surgery >30 minutes, age ≥3 years, strabismus surgery, and history of POV or PONV in relatives with 1(one) point against each factor [43] are used (Figure 3).

Despite the limitations in accuracy of PONV risk scoring system, their use to better tailor antiemetic interventions has been shown to significantly reduce the incidence of PONV in general and particularly in high risk patient populations, while avoiding the expense and potential side effects of prophylactic antiemetics in lower-risk individuals [44].

## 9. Intraoperative Anaesthetic Management

 To decrease the incidence of PONV without compromising on analgesia, regional anaesthesia is to be used whenever possible to minimise the intake of opioids. Emetogenic induction agents like nitrous oxide, inhalational agents, and etomidate and ketamine are avoided in patients with serious risk of PONV. Other strategies may be to supplement analgesia with NSAIDs and regional anaesthesia to decrease the usage of perioperative opioids. Anticholinesterases like neostigmine should always be used in appropriate dosage after confirming reversal characteristics with use of neuro-muscular monitoring.

Other strategies may be to use total intravenous anaesthesia (TIVA) with propofol, prevention of hypotension, adequately hydrate and oxygenate the patient, and sedate an anxious patient taking a multimodal approach to PONV management. Some studies have stated that nonpharmacologic strategies like acupuncture reduce the incidence of PONV. These strategies are summarized in Table 1.

## 10. Currently Available Antiemetics [45]

There are at least four major receptor systems involved in the aetiology of PONV. Currently, available antiemetics may act at the cholinergic (muscarinic), dopaminergic (D2), histaminergic (H1), or serotonergic (5HT3) receptors. Neurokinin-1(NK-1) receptor antagonists are also being investigated. Cholinergic receptors are found in the vomiting center and vestibular nuclei. The area postrema is rich in dopamine (D2), opioid, and serotonin (5HT_3_) receptors. The nucleus tractus solitaries is rich in enkephalins and in histaminic (H1), muscarinic cholinergic, and NK-1 receptors. the latter are also found in the dorsal motor nucleus of the vagus nerve. 

Ondansetron, granisetron, dolasetron, tropisetron, and other serotonin antagonists have been shown to provide effective treatment and prophylaxis of PONV and are associated with a low incidence of side effects. These agents are not dopamine, muscarinic, or histamine receptor antagonists and, as such, are not associated with the side effects common to those classes. Side effects common to the serotonin antagonists include headache, lightheadedness, dizziness, and constipation.

Metoclopramide acts on both central dopamine and serotonin receptors, and has both prokinetic and antiemetic effects. Metoclopramide increases gastrointestinal tract motility, decreases gastric emptying time and gastric volume, increases lower esophageal sphincter tone, and is usually well tolerated in adults. Extrapyramidal effects and dystonia are more often seen in the pediatric population.

Dexamethasone is an effective antiemetic though its mechanism of action remains uncertain. Most likely mechanisms are prostaglandin inhibition in peripherally, with facilitation of serotonergic antagonism and endorphin release centrally. Its long duration of action and cost effectiveness make dexamethasone an attractive choice in the PONV management.

Droperidol is a butyrophenone effective in the treatment of PONV. Like the phenothiazines, droperidol acts competitively on central dopaminergic receptors and is associated with sedation, lethargy, agitation, and extrapyramidal effects. The “black box” warning to the droperidol drug information sheet by the FDA has reduced the use of droperidol to that of rescue agent for intractable cases of PONV. A prolonged QTc interval develops in some patients putting them at risk to develop torsade de pointes. Despite the limited evidence that antiemetic doses trigger this dangerous arrhythmia, electrocardiographic monitoring remains mandatory whenever this drug is being used, and needs to be used for 2 to 3 hours after its use.

Ephedrine and other agents that help maintain blood pressure may be used to prevent the nausea associated with hypotension postoperatively.

Promethazine, prochlorperazine, and chlorpromazine are phenothiazines that exert their antiemetic effects by directly acting on the central dopaminergic receptors of the chemoreceptor trigger zone. These agents are most effective in the treatment of opioid-induced PONV, but their use as the primary treatment for PONV is limited by their tendency to cause sedation.

Scopolamine is an anticholinergic agent that acts on the muscarinic and histaminic receptors of the vestibular apparatus and the nucleus of the tractus solitarus to reduce the incidence of PONV. It has been found to be very effective in patients treated with opioids for postoperative pain control and after middle ear surgery, though use is limited by a high incidence of sedation and dry mouth. Scopolamine, in the form of a transdermal patch applied the evening before or the morning of surgery, has been shown to reduce the incidence of PONV as effectively as ondansetron. It is effective if applied the evening before surgery or 4 h before the end of anesthesia due to its 2–4 h onset of effect. 

Placement of capsaicin ointment on the K-D2 point (the Korean hand acupressure point in Koryo Hand Therapy) of both hands 1 h before laparoscopic cholecystectomy resulted in a significantly lower incidence of PONV, and the need for rescue antiemetic treatment was also lower. Stimulation of the P6 acupressure point has been associated with decreased postoperative nausea and vomiting in high risk women and has also been shown to increase patient tolerance to experimental nauseogenic stimuli, as well as reducing the number of symptoms experienced.

Current pharmacotherapies available for the management of PONV and POV are summarized in Tables 2 and 3.

## 11. Recommended Strategy for PONV Prophylaxis

The risk of PONV should be estimated for each patient. No prophylaxis is recommended for patients at low risk for PONV except if they are at risk for medical consequences from vomiting, for example, patients with wired jaws or increased intracranial pressure or who are having fundoplications surgery. For patients at moderate to high risk for PONV, regional anaesthesia should be considered. If this is not possible or contraindicated and a general anaesthesia is used, strategies to minimize the baseline risk of PONV should be adopted. The use of combination antiemetic therapy and more appropriately a multimodal approach includes use of two or more interventions. A multimodal approach to minimize PONV combines pharmacologic and nonpharmacologic prophylaxis as well as interventions that reduce baseline risk.

In general, combination therapy is superior to monotherapy for PONV prophylaxis. Drugs with different mechanism of action should be used in combination to optimize efficacy [46–48].

## 12. Recommended Strategy for PONV Who Did Not Receive Prophylaxis or in Whom Prophylaxis Failed

When persistent nausea and vomiting occur after the patient has left the postanaesthesia care unit, the first response should be a bedside examination to exclude an initiating medication or mechanical factor. Contributing factor might include patient-controlled analgesia with morphine, blood draining down the throat, or an obstruction of the gut. Once medication and mechanical factors are excluded, rescue antiemetic therapy can be initiated.

If a patient has received no prophylaxis, therapy with small dose 5HT3 receptor antagonists should be initiated on the first sign of PONV. Small-dose therapy includes ondansetron 1 mg, dolasetron 12.5 mg, granisetron 0.1 mg, and tropisetron 0.5 mg. For all other antiemetics, data on their therapeutic efficacy are sparse, and optimal doses are unknown [49]. 

When prophylaxis with dexamethasone fails to prevent PONV, treatment within a small dose 5-HT_3_ receptor antagonist has been recommended [42]. When prophylaxis with a 5-HT_3_ antagonist is inadequate to prevent PONV, a 5-HT_3_ antagonist should not be initiated as rescue therapy within the first 6 hours after surgery because it confers no additional benefit [50]. Similarly, the failure of prophylaxis with a 5-HT_3_ antagonist plus dexamethasone should be treated with a drug from another class, for instance, droperidol or promethazine.

A triple-therapy dosing regimen (for instance, a 5-HT_3_ antagonist, droperidol, and dexamethasone) has never been tested. If the patient experiences PONV despite triple prophylaxis, the triple regimen should not be repeated within the first 6 hours of administration, and an alternative antiemetics should be administered. Propofol 20 mg as needed can be considered for rescue therapy in patients still in the postanaesthesia care unit [51]. The antiemetic effect with small doses of propofol is probably brief.

When PONV occurs more than 6 hours after surgery, repeat dosing of 5-HT_3_ antagonists and droperidol can be considered. The optimal dose and interval for readministration of these two antiemetics remain unknown.

## 13. Combination Antiemetic Therapy

None of the available antimetics is entirely effective for preventing PONV, especially in high-risk patients. Since at least four major receptor systems are involved in the aetiology of PONV, a better prophylaxis might be achieved by using a combination of agents acting at different receptor sites. For example, if the serotonin receptors have already been blocked, consider adding an anticholinergic, antidopaminergic, or antihistamine. The concept of combination antiemetic therapy was first introduced in chemotherapy induced vomiting. Its success prompted similar research in the field of PONV [46, 47].

The most commonly studied combinations have included a 5-HT3 receptor antagonist with either droperidol or dexamethasone. Both combination regimens appear to be equally efficacious [46–48]. 

Combination therapy is most cost effective for patients at high risk for the development of PONV, and medium risk patients are often successfully treated with a single agent. Increasing the number of antiemetics administered reduced the incidence of postoperative nausea and vomiting from 52 percent when no antiemetics were used to 37 percent, 28 percent, and 22 percent when one, two, and three antiemetics, respectively, administered, which corresponds to a 26 percent reduction in the relative risk of nausea and vomiting for each additional antiemetic used [14].

## 14. Multimodal Approach [52]

In addition to using a combination of antiemetics acting at different receptor sites, the multifactorial aetiology of PONV might be better addressed by the adoption of multimodal approach.

A multimodal approach to minimize PONV combines nonpharmacologic and pharmacologic prophylaxis as well as interventions that reduce baseline risk [1, 50, 51]. Scuderi et al. [50] tested the efficacy of a multimodal approach to reducing PONV. Their multimodal approach consisted of preoperative anxiolysis and aggressive hydration (25 mL/Kg), oxygen, prophylactic antiemetics (droperidol, 0.625 mg and dexamethasone, 10 mg at induction and ondansetron, 1 mg at end of surgery) total intravenous anaesthesia with propofol and remifentanil, and ketorolac (30 mg). No nitrous oxide or neuromuscular blockade was used. Patients who received multimodal therapy had a 98% complete response rate (no PONV and no rescue antiemetic) compared with 76% response rate among patients receiving antiemetic monotherapy and a 59% response rate among those receiving routine anaesthetic plus saline placebo.

## 15. Novel Antiemetics

### 15.1. Neurokinin-1 Antagonists

Substance P, a member of the tachykinin family of neuropeptides is an important neurotransmitter in afferent pathways of emesis [52]. Substance P may be released from enterochromaffin cells in the stomach and intestine (e.g., postoperative trauma) or from sensory neurons (e.g., radiation, chemotherapeutic agents) [53]. The NKI receptors are located in the area postrema and are thought to play a particularly important role in emesis. However, NK1 receptor antagonists (NK1 RAS) are thought to exert their mechanism of action on neurons in the “afferent relay station” situated between the medial neurotransmitter system and the central pattern generator for vomiting [53] although this has not been definitely isolated for humans. The potential NK1 receptor blocking activity located deeper in the brain stem is thought to prevent both acute and delayed emesis, whereas 5-HT_3_ receptor antagonists are largely effective only against acute emesis [53], leading to considerable recent interest in the use of NK1 receptor antagonists for prophylaxis of PONV.

Aprepitant is the only NK1 receptor antagonist currently approved by the FDA for the prophylactic management for PONV. It is available in oral capsule in 40 mg to be administered between 1–3 hours before surgery. It has a long half-life of about 48 hours [54]. It appears to have better efficacy in the prevention of PONV when compared with ondansetron [55].

### 15.2. Long-Acting Serotonin Antagonist

Palonosetron has the longest elimination half-life of all the currently available serotonin antagonists at about 40 hours [53]. Its long duration of action can also be explained by its high binding affinity for 5-HT_3_ receptors [54]. It is 62% bound to plasma proteins [56].

The liver metabolizes approximately 50% of palonosetron. The two primary metabolites, N-oxide-palonosetron and 6-(s)-hydroxyl-Palonosetron, are essentially inactive [56].

## 16. Suggested Regimen

Once the patient is placed into the proper risk group, then specific recommendations can be made regarding proper care.

1. No prophylaxis is recommended for patients at lowrisk for PONV except if they are at risk for medical consequences from vomiting, for example, patients with wired-jaw.
2. In moderate risk patients, if prophylactic dose of dexamethasone fails, then a serotonin antagonist should be used as soon as nausea or vomiting occurs. If treatment with this single agent fails, then aggressive and properly chosen combination therapy should be utilized. However, the best available combination and the optimum doses of antiemetic agents when used in combination are yet to be established.
3. In high-risk patients, dexamethasone plus a serotonin antagonist should be utilized for prophylaxis. If this prophylaxis fails, aggressive and properly chosen combination therapy should be utilized.
4. There is paucity of data on the use of antiemetics for the treatment of PONV in patients who failed prophylaxis or did not receive prophylaxis. This is due to the difficulty in performing such studies since a large number of patients would need to be recruited in order to obtain the required target of patients who eventually experience PONV.

The 5-HT_3_ receptor antagonists were the most commonly tested drugs in rescue clinical trials. Similar to their use in PONV prophylaxis, the antivomiting efficacy of the 5-HT_3_ receptor antagonists is more pronounced than their antinausea efficacy. There is no evidence of dose responsiveness for those agents when used for rescue. Therefore, small doses of these agents have been recommended for treatment: ondansetron 4 mg, granisetron 0.1 mg, dolasetron 12.5 mg, and tropisetron 0.5 mg.

## 17. Conclusion

Without prophylactic intervention, PONV will develop in an estimated one third of patients (range, 10% to 80%) who undergo inhalational anaesthesia. The consequences of PONV include delayed discharge from the PACU, unanticipated hospital admission, increased incidence of pulmonary aspiration, and significant postoperative discomfort. The ability to identify high-risk patients for prophylactic intervention can significantly improve the quality of patient care and satisfaction in the PACU.

Therefore, depending upon the level of risk, prophylaxis should be initiated with monotherapy or combination therapy [8]. All prophylaxis in children at moderate or high risk for postoperative vomiting should include combination therapy using a 5-HT3 antagonist and a second drug from other class. Because the effects of interventions from different drug classes are additive, combining interventions has an additive effect in risk reduction.

When rescue therapy is required, the antiemetic should be chosen from a different therapeutic class than the drugs used for prophylaxis.

## Figures and Tables

**Table 1:** Anaesthetic strategies to decrease PONV.

(1) Use of regional anaesthesia.
(2) Avoid emetogenic stimuli
(a) Nitrous oxide
(b) Inhalational agents
(c) Etomidate and Ketamine.
(3) Minimize the following:
(a) Intraoperative and postoperative opioids
(b) Adequate analgesia incorporating local anaesthetics, NSAIDs, and opioid as required
(c) Limiting the dose of neostigmine to 2.5 mg in adults.
(4) To consider the following:
(a) Total intravenous anaesthesia (TIVA) with propofol
(b) Adequate hydration, especially with colloids.
(c) Use of intraoperative supplemental oxygen
(d) Use of a anxiolytics, for example, benzodiazepines
(e) Nonpharmacological techniques, for example, acupuncture.

**Table 2:** Antiemetic doses and timing for administration in adults.

Drug	Dose	Timing
Ondansetron	4–8 mg IV	At end of surgery
Dolasetron	12.5 mg/IV	At end of surgery
Granisetron	0.35–1mg IV	At end of surgery
Tropisetron	5 mg IV	At end of surgery
Dexamethasone	5–10 mg IV	At induction
Droperidol	0.625–1.25 mg IV	At end of surgery
Ephedrine	1–2 mg/KG IV	At end of surgery
Prochlorperazine	5–10 mg IV	At end of surgery
Promethazine	12.5–25 mg IV	At end of surgery
Scopolamine	Transdermal patch	Applied prior evening or 4 hr before end of surgery

Based on the original article by Gan et al. [52].

**Table 3:** Antiemetic doses for prophylaxis of postoperative vomiting (POV) in children.

Drug	Dose
Dexamethasone	150 *μ*g/Kg up to 5 mg
Dimenhydrinate	0.5 mg/Kg up to 25 mg
Dolasetron	350 *μ*g/kg up to 12.5 mg
Droperidol^a^	10–15 *μ*g/Kg up to 1.25 mg
Granisetron	40 *μ*g/Kg up to 0.6 mg.
Ondansetron^b^	50–100 *μ*g/Kg up to 4 mg.
Perphenazine	70 *μ*g/Kg up to 5 mg
Tropisetron	0.1 mg/Kg up to 2 mg.

^a^See food and drug administration (FDA) “black box” warning.

Recommended doses 10 to 15 *μ*g/Kg.

^b^Approved for POV in paediatric patients aged one month and older.

Reproduced from the original article by Gan et al. [52].
